# Unraveling glioblastoma diversity: Insights into methylation subtypes and spatial relationships

**DOI:** 10.1093/noajnl/vdae112

**Published:** 2024-06-28

**Authors:** Martha Foltyn-Dumitru, Haidar Alzaid, Aditya Rastogi, Ulf Neuberger, Felix Sahm, Tobias Kessler, Wolfgang Wick, Martin Bendszus, Philipp Vollmuth, Marianne Schell

**Affiliations:** Department of Neuroradiology, Heidelberg University Hospital, Heidelberg, Germany; Section for Computational Neuroimaging, Department of Neuroradiology, Heidelberg University Hospital, Heidelberg, Germany; Department of Neuroradiology, Heidelberg University Hospital, Heidelberg, Germany; Department of Neuroradiology, Heidelberg University Hospital, Heidelberg, Germany; Section for Computational Neuroimaging, Department of Neuroradiology, Heidelberg University Hospital, Heidelberg, Germany; Department of Neuroradiology, Heidelberg University Hospital, Heidelberg, Germany; Department of Neuropathology, Heidelberg University Hospital, Heidelberg, Germany; Clinical Cooperation Unit Neuropathology, German Cancer Consortium (DKTK), German Cancer Research Center (DKFZ), Heidelberg, Germany; Department of Neurology and Neurooncology Program, Heidelberg University Hospital, Heidelberg University, Heidelberg, Germany; Clinical Cooperation Unit Neurooncology, German Cancer Research Center (DKFZ), Heidelberg, Germany; Department of Neurology and Neurooncology Program, Heidelberg University Hospital, Heidelberg University, Heidelberg, Germany; Clinical Cooperation Unit Neurooncology, German Cancer Research Center (DKFZ), Heidelberg, Germany; Department of Neuroradiology, Heidelberg University Hospital, Heidelberg, Germany; Department of Neuroradiology, Heidelberg University Hospital, Heidelberg, Germany; Section for Computational Neuroimaging, Department of Neuroradiology, Heidelberg University Hospital, Heidelberg, Germany; Department of Neuroradiology, Heidelberg University Hospital, Heidelberg, Germany; Section for Computational Neuroimaging, Department of Neuroradiology, Heidelberg University Hospital, Heidelberg, Germany

**Keywords:** DNA methylation subtypes, glioblastoma, MRI, support vector regression-based lesion-symptom mapping

## Abstract

**Background:**

The purpose of this study was to elucidate the relationship between distinct brain regions and molecular subtypes in glioblastoma (GB), focusing on integrating modern statistical tools and molecular profiling to better understand the heterogeneity of Isocitrate Dehydrogenase wild-type (IDH-wt) gliomas.

**Methods:**

This retrospective study comprised 441 patients diagnosed with new IDH-wt glioma between 2009 and 2020 at Heidelberg University Hospital. The diagnostic process included preoperative magnetic resonance imaging and molecular characterization, encompassing IDH-status determination and subclassification, through DNA-methylation profiling. To discern and map distinct brain regions associated with specific methylation subtypes, a support-vector regression-based lesion-symptom mapping (SVR-LSM) was employed. Lesion maps were adjusted to 2 mm³ resolution. Significance was assessed with beta maps, using a threshold of *P* < .005, with 10 000 permutations and a cluster size minimum of 100 voxels.

**Results:**

Of 441 initially screened glioma patients, 423 (95.9%) met the inclusion criteria. Following DNA-methylation profiling, patients were classified into RTK II (40.7%), MES (33.8%), RTK I (18%), and other methylation subclasses (7.6%). Between molecular subtypes, there was no difference in tumor volume. Using SVR-LSM, distinct brain regions correlated with each subclass were identified: MES subtypes were associated with left-hemispheric regions involving the superior temporal gyrus and insula cortex, RTK I with right frontal regions, and RTK II with 3 clusters in the left hemisphere.

**Conclusions:**

This study linked molecular diversity and spatial features in glioblastomas using SVR-LSM. Future studies should validate these findings in larger, independent cohorts to confirm the observed patterns.

**Key Points**

SVR-LSM reveals spatial differences between DNA methylation-based subclasses of GB.
MES and RTK II subtypes are linked to left-hemispheric regions.
RTK I subtype is associated with the right frontal regions.

**Importance of the Study**

The importance of this study lies in its potential to deepen our understanding of GB pathophysiology by elucidating the relationship between its molecular diversity and spatial distribution within the brain. By identifying preferential localization of GB methylation subtypes, particularly within critical brain regions such as the insula and frontal lobes, the study sheds light on the role of the brain’s distinct metabolic and functional environments in tumor development and progression contributing to the refinement of GB treatment strategies.

Glioblastoma (GB), the most prevalent type of primary brain tumor, presents a significant clinical challenge due to its diffuse infiltration into surrounding brain tissue and its starkly heterogeneous nature, both molecularly and clinically. Despite improvements in understanding and treatment, the prognosis for GB patients remains sober, with a 5-year survival rate hovering around 5%, and considerable variability in individual outcomes.^1^ Factors such as functional level, age, tumor size, and location play a pivotal role in clinical decision-making, especially in presurgical planning where the extent of resection is often correlated with survival outcomes.^2–4^

Since 2016, the World Health Organization (WHO) classification of CNS tumors has included molecular parameters alongside histological characteristics to better categorize this diverse disease.^5,6^ This molecular stratification is essential as each glioma subtype may originate from distinct cells, with early events in gliomagenesis being particularly critical for the development of targeted therapies. Recent studies and advancements in technologies like molecular profiling have significantly contributed to our understanding of gliomagenesis, indicating correlations between tumor location, molecular subtypes, and clinical outcomes.^7–10^ For instance, Tejada Neyra et al. demonstrated that IDH-mutated gliomas tend to cluster around the rostral extension of the lateral ventricles using a voxel-based lesion-symptom mapping.^7^ Additionally, Ellingson et al. found that EGFR-amplified and EGFR variant 3-expressing tumors predominantly occurred in the left temporal lobe. In contrast, tumors without PTEN loss were mostly found in the frontal lobe.^10^

Current improvements in global DNA methylation profiling, which utilizes arrays to analyze DNA methylation patterns across the genome, have enhanced the precision in molecularly classifying central nervous system (CNS) tumors.^11,12^ This technique goes beyond traditional clinicopathological assessments, offering a more detailed stratification of patients. With this DNA methylation-based classification, GB can be categorized into several subclasses, such as receptor tyrosine kinase (RTK) I, II, III, H3.3 G34-mutant, midline, MYCN, and mesenchymal (MES).^11,13^ Among adults, RTK I, RTK II, and MES are the most prevalent methylation subclasses. In patient groups primarily involved in clinical trials, these 3 subclasses showed similar survival outcomes.^14–16^ However, recent research has highlighted clinical and therapeutic differences among these subclasses: Wick et al. revealed that the prognostic benefit of MGMT promoter methylation might predominantly apply to the RTK II subclass in cases treated with temozolomide monotherapy.^14^ Moreover, Drexler et al. observed a distinct survival advantage linked to the maximal extent of resection in RTK I and RTK II subclasses, a trend that was not present in the MES subclass.^17^ Additionally, Ricklefs et al. noted a strong correlation between the RTK II subclass and both preoperative and long-term seizures.^18^ These studies collectively underscore the importance of subclass-specific approaches in the management and treatment of GB.

The present study aims to investigate the preferential localizations of various GB methylation subtypes within the brain using support vector regression-based lesion-symptom mapping (SVR-LSM) to elucidate the intricate relationship between the molecular diversity of GB and its spatial distribution.

## Materials and Methods

### Dataset

Ethical clearance was granted by the University of Heidelberg’s Ethics Committee, which also provided a waiver from informed consent obligations (Ethics Approval No. S-784 2018) for this study. The cohort encompassed 441 patients, diagnosed with Isocitrate Dehydrogenase wild-type (IDH-wt) glioma^6^ at a time frame from 2009 to 2020. Criteria for inclusion involved 2 key aspects: (1) access to crucial molecular data from tissue samples acquired at the time of the initial surgery at the Department of Neurosurgery at Heidelberg University Hospital, and (2) the availability of standardized preoperative MRI data, collected at the Department of Neuroradiology at Heidelberg University Hospital.

### MRI Examinations and Tumor Segmentation

Image acquisition was performed using standard clinical protocols on a 3 Tesla MRI system (Magnetom Verio, TIM Trio, or Skyra by Siemens Healthcare) and utilized a 12-channel head-matrix coil. In line with globally recognized imaging guidelines^19^, our methodology encompassed the collection of T1-weighted images both before (T1) and after (cT1) the administration of a gadoterate meglumine bolus (0.1 mmol/kg, Dotarem, Guerbet), in addition to acquiring axial 2D FLAIR and T2-weighted images.

For tumor segmentation, we employed a nnUNET-modified version of the HD-GLIO application, which leverages advanced deep learning techniques, to accurately identify different tumor compartments such as necrotic, contrast-enhanced, and non-contrast-enhanced T2/FLAIR hyperintense areas.^20,21^ The results of the automated segmentation underwent review by MF, a neuroradiology resident with 6 years of experience. Corrections were required in 59/423 (13.95%) cases.

Finally, we integrated the distinct segmentation masks—those identifying contrast-enhancing, non-contrast-enhanced T2/FLAIR hyperintense, and necrotic tumor components—into a unified segmentation mask for comprehensive analysis.

#### DNA Methylation Profiling

.—Genomic DNA was isolated from tumor samples and subjected to comprehensive analysis for DNA methylation patterns across the genome, employing either Illumina 450k/850k or EPIC arrays. The methodology for processing the DNA methylation data involved unique, customized techniques, as detailed in prior publications.^13,22^ For classifying the samples, the brain tumor classification system developed by the German Cancer Research Center (Deutsches Krebsforschungszentrum, DKFZ) version v11b4 has been used.^11^

#### Support Vector Regression-Based Lesion-Symptom Mapping (SVR-LSM)

.—SVR-LSM analyses were conducted utilizing Matlab (version R2020a) and the SVR-LSM toolbox (v0.15, released on June 16, 2019), a tool created by DeMarco and Turkeltaub in 2018,^23^ which builds upon methodology proposed by Zhang and colleagues in 2014.^24^ This technique employs a multivariate analysis approach, utilizing machine learning-driven multiple regression analysis to investigate the relationship between lesioned voxels and methylation subtypes, while concurrently considering the lesion condition of all voxels. Separate analyses were conducted for each methylation type to discern their spatial distributions. Since MES, RTK I, and RTK II represent most GB methylation types, our SVR-LSM analysis focused on these 3 subtypes. Each subtype was individually analyzed against the rest of the cohort. While other methylation types were not excluded, they were consistently included in the comparison group for MES, RTK I, and RTK II analyses.

In this study, all precontrast T1-weighted images were aligned to the FSL (FMRIB Software Library) 1 mm MNI template (https://fsl.fmrib.ox.ac.uk/fsl/fslwiki/Atlases). This alignment process was facilitated using a customized version of the fsl_anat function, which integrates both linear and nonlinear registration techniques, alongside an additional feature for tumor area masking (as provided by the FMRIB software library, FSL,^25^ accessible at http://fsl.fmrib.ox.ac.uk/fsl/fslwiki/FSL). Following the registration step, the transformation matrices were applied to the tumor masks.

The analysis concentrated on voxels that were affected in at least 10 patients.^26^ This criterion was set to ensure the findings were broadly applicable and not biased by voxels impacted in only a few patients. No additional exclusion criteria related to localization were applied. Images were resampled to a resolution of 2 mm³, and lesion volume correction was meticulously performed using a regression model that accounted for both methylation profile and voxels, with residuals utilized in the model. To determine the optimal SVR hyperparameters for our dataset, a Bayesian optimization approach with 200 iterations, implemented in Matlab (bayesopt), was employed. Subsequently, SVR was executed with a 5-fold cross-validation using the optimized hyperparameters alongside 10 000 permutations and a voxelwise threshold of *P* < .005 to identify voxels that were significantly associated with DNA methylation profiles. Each fold of the cross-validation utilized a distinct subset of the lesion and methylation data from 80% of the patients for training the SVR model, while the data from the remaining 20% were reserved for model testing. The 5 resulting beta value maps were then averaged to produce a conclusive map of average voxelwise beta values. Further refinement of the permutation-thresholded SVR maps was achieved by applying a minimum cluster extent criterion of 100 contiguous voxels.

Identification of significant voxels’ brain regions utilized the Hammersmith atlas,^27,28^ an adult brain maximum probability atlas based on 83 manually delineated regions.

### Statistical Analysis

Analyses of the data were performed utilizing R software (Version 4.2.2, provided by the R Foundation for Statistical Computing). Continuous variables were expressed using either mean values along with their standard deviations (SD) or median values accompanied by interquartile ranges. In the case of categorical variables, these were reported as number counts and their respective percentages. To compare continuous variables, the study applied the Wilcoxon–Mann–Whitney test, whereas the chi-squared tests were used for the analysis of categorical data.

In this research, statistical significance was determined by setting the *P*-value cutoff at less than 0.05 for all conducted analyses.

## Results

### Patient Cohort

**Figure 1.**
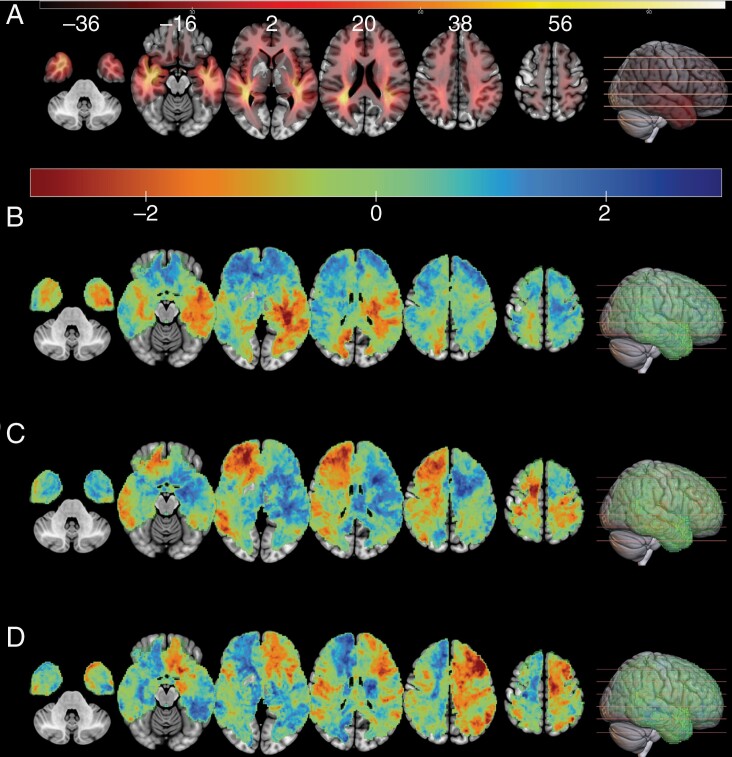
Overlay of all tumor masks limited to minimum lesion cutoff at *n* = 10 (A). Unthresholded voxelwise *z*-maps for the distinct methylation subclasses: MES (B), RTK I (C), and RTK II (D). Warmer colors (red and orange), representing voxels with lower *z*-scores, indicate a higher occurrence of specific DNA methylation types in certain regions. Overlay on mni152 template. “MNI” refers to the Montreal Neurological Institute. The term “MNI template” denotes a standard anatomical coordinate system for the human brain, developed by the Montreal Neurological Institute, which is widely used in neuroimaging to ensure consistency and comparability across different studies.^29^

A total of 441 patients with newly diagnosed GB were initially considered for the study. Out of these, 423 (95.9%) patients met the inclusion criteria and were thus enrolled in the investigation. Twelve patients were excluded due to missing high-resolution T1w images, and 6 patients lacked sufficient voxels meeting the minimum lesion cutoff (*n* = 10). Of the 204 893 voxels containing any lesions, 68% (139 519 voxels) met the minimum overlap criterion (*n* = 10) Figure 1A.

Following DNA methylation profiling, patients were classified into methylation subclasses. Predominantly, RTK II was observed (*n* = 172, 40.7%), followed by MES (*n* = 143, 33.8%), RTK I (*n* = 76, 18%), and others (*n* = 32, 7.6%). The group of others was composed as follows: GBM, MID (*n* = 9), DMG, K27 (*n* = 9), GBM, G34 (*n* = 2), LGG, DNT (*n* = 2), LGG, PA/GG ST (*n* = 2), PXA (*n* = 2), GBM, MYCN (*n* = 1), LGG, RGNT (*n* = 1), LGG, GG (*n* = 1), LGG, MYB (*n* = 1), ANA PA (*n* = 1), and HEMI (*n* = 1). Detailed demographic data, MGMT status and image characteristics of the MES, RTK I, and RTK II groups are provided in Table 1. Survival data were not available for 2 patients, 1 from the MES group and 1 from the RTK I group.

**Table 1.** Demographic, MGMT Status, and Volumetric Tumor Information of the Included Patients

Parameter	MES	RTK I	RTK II	P-value
**Clinical**				
Number of patients	143	76	172	NA
Age in years [mean ± SD]	61 ± 12	63 ± 11	64 ± 11	.37
Gender [% female]	49	33	47	.06
Survival in months [mean ± SD]	16 ± 16	16 ± 19	16 ± 13	.44
**Molecular**				
MGMT (methylated [n(%)])	61 (43)	34 (45)	80 (47)	*.*79
**Volumes [in cm** ^**3**^ **]**				
Whole tumor [mean ± SD]	87 ± 60	89 ± 61	82 ± 59	.65
Non-contrast enhancing T2/FLAIR [mean ± SD]	63 ± 48	56 ± 44	54 ± 40	.30
Contrast enhancement [mean ± SD]	17 ± 18	23 ± 22	20 ± 20	.09
Necrosis [mean ± SD]	7.2 ± 11	11 ± 15	8.2 ± 12	.09

### Volumetric and SVR-LSM Results

With a mean size of 89 cm^3^, tumors in the RTK I group were the largest, followed by the MES group with a size of 87 cm^3^ and the smallest in the RTK II group with 82 cm^3^, with no significant difference between the different classes (*P* = .65). The volumetric analysis of the individual tumor compartments, non-contrast enhancing T2/FLAIR, contrast-enhancing, and necrotic parts, also showed no significant difference between the different methylation classes, with *P* ≥ .09 each (Table 1).

Unthresholded voxelwise *z*-score maps (Figure 1B–D) offer a comprehensive visualization of DNA methylation distribution patterns across brain regions. Lower *z*-scores denote a heightened prevalence of specific methylation types in certain areas, thereby highlighting regions where these methylation patterns are particularly prominent, serving the exploratory purpose of identifying potential areas of interest for further investigation.

**Figure 2.**
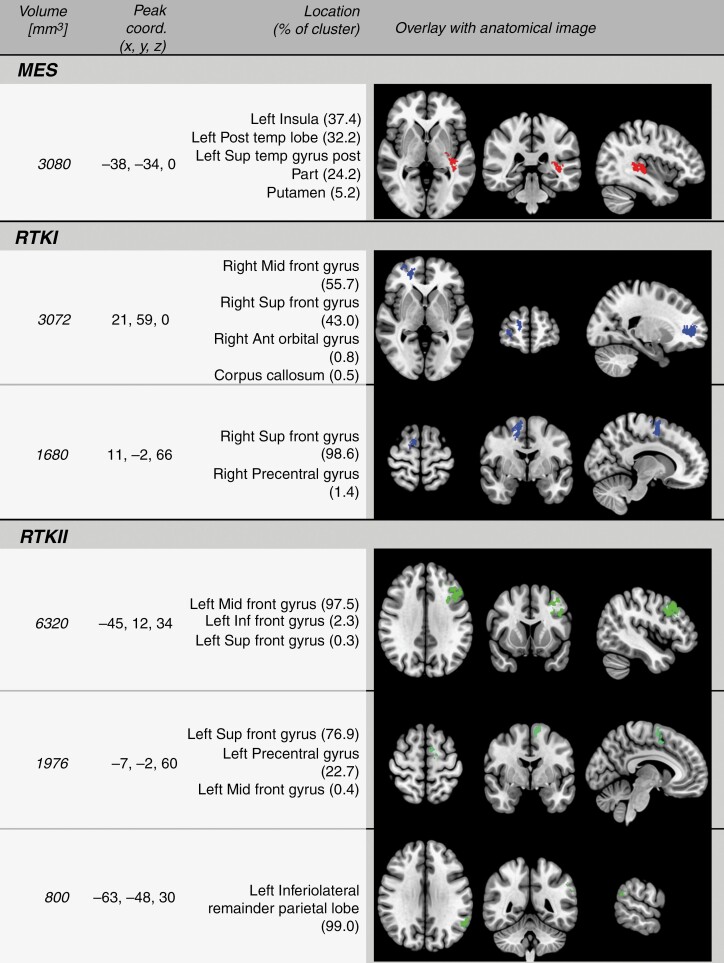
Summary of the identified clusters for the 3 different methylation classes. Anatomical location based on Hammersmith atlas. Abbreviation: Post: posterior; sup:superior; ant: anterior; inf: inferior; mid: middle; temp: temporal; front: frontal.

The SVR-LSM analysis identified a tumor location predilection for the MES subgroup with 1 left hemispheric cluster, which included the insula (37.4% of the cluster), posterior temporal lobe (32.2%), superior temporal gyrus posterior part (24.2%), and putamen (5.2%). The RTK I subgroup showed 2 clusters: 1 in the middle frontal brain parenchyma encompassing right middle frontal gyrus (55.7% of the cluster), right superior frontal gyrus (43.0%), right anterior orbital gyrus (0.8%), and corpus callosum (0.5%). The other cluster included the right superior frontal gyrus (98.6% of the cluster) and the right precentral gyrus (1.4%). RTK II exhibited 3 left hemispheric clusters: 1 encompassing left middle frontal gyrus (97.5% of the cluster), inferior frontal gyrus (2.3%), and superior frontal gyrus (0.3%), 1 including superior frontal gyrus (76.9%), precentral gyrus (22.7%), and middle frontal gyrus (0.4%) and a third cluster in the inferolateral remainder parietal lobe (99.0% of the cluster) (Figures 1 and 2).

## Discussion

Advancements in global DNA methylation profiling have significantly enhanced the molecular classification of CNS tumors, facilitating a more detailed patient stratification beyond traditional clinicopathological methods. This classification identifies several GB subclasses, including RTK I, RTK II, and MES, each exhibiting distinct clinical and therapeutic responses, underscoring the need for subclass-specific approaches in GB management and treatment. In this study, we identified preferred brain regions for various GB methylation subclasses using SVR-LSM, bridging the gap between molecular heterogeneity and spatial characteristics.

Several studies reported preferential localization for molecular GB subtypes using various methods. Tejada Neyra et al. demonstrated that IDH-mutated gliomas tend to cluster around the rostral extension of the lateral ventricles, utilizing voxel-based lesion-symptom mapping.^7^ Similarly, Ellingson et al. identified that EGFR-amplified tumors predominantly occur in the left temporal lobe, whereas tumors without PTEN loss are mostly located in the frontal lobe.^10^ These findings underscore the importance of considering the spatial distribution of molecular subtypes in GBs.

Within the MES subgroup, we observed a distinct tumor localization preference characterized by a cluster in the left hemisphere that included the left insula and temporal lobe. While this observation may align with existing literature on the role of the insula in modulating immune responses, it is important to approach this hypothesis with caution. Pivoting work by Ramirez-Amaya highlighted the link between insular damage and impaired acquisition of conditioned immunosuppression in rats over 2 decades ago.^30^ Subsequent research has indicated heightened insular activity and altered functional connectivity under pro-inflammatory conditions induced by vaccine administration.^31^ Additionally, research by Dejaegher et al. explored the correlation between epigenetic GB subgroups, immune cell infiltration, systemic immune alterations during chemoradiotherapy, and patient outcomes in a cohort of 93 individuals. This research revealed that tumors within the mesenchymal category exhibited the highest concentration of CD3 + T cells.^16^ Although these insights are intriguing, further research is needed to confirm whether the immune activity associated with mesenchymal tumors can be attributed to their localization within the insula.

For the RTK I group we observed 2 predilection clusters in the right frontal lobe, consistent with existing studies reporting a higher prevalence of proneural tumors, including those within the RTK I methylation class, in frontal lobe regions.^10^

In our investigation of the RTK II class, we identified 3 distinct clusters indicative of tumor predilection: 2 located within the left frontal region and a smaller one in the left parietal area. Previous research has emphasized the evolutionary specialization of the prefrontal cortex to manage elevated levels of glutamate neurotransmission, a pivotal element in augmenting human cognitive functions. This adaptation has led to the formation of a metabolic niche within this region of the brain.^32,33^ In their comprehensive study, Ricklefs et al. analyzed a cohort of *n* = 111 GB patients who underwent surgical treatment, focusing on the correlation between the tumor’s methylation profile and the occurrence of epileptic seizures. Their findings revealed that the RTK II methylation subclass serves as a significant predictor of seizure risk, both before and after surgery.^18^ Furthermore, they showed, that this subclass is characterized by the upregulation of genes involved in vesicle and glutamate trafficking. Many studies suggest that the neurotransmitter glutamate plays a central role in this context and supports both the development of epilepsy and tumor progression.^34–37^ In IDH wt GBs, dysregulated expression of glutamate receptor genes results in overproduction of glutamate. This excess glutamate not only stimulates the proliferation and dissemination of glioma cells^34,35^ but also triggers epileptic episodes.^36,37^

Tejada Neyra et al. investigated the association between tumor localization and the 3 methylation classes RTK I, RTK II, and MES in *n* = 237 patients with newly diagnosed glioblastoma using voxel-based lesion-symptom mapping (VLSM).^7^ Interestingly, they did not observe any tumor localization predilection for the different methylation classes. However, our work differs in 2 critical methodological aspects. Unlike Tejada Neyra et al. we used additional non-linear image registration. Non-linear methods, in contrast to linear methods alone, are better equipped to handle distortions and deformations in tissues, particularly in scenarios, where tumor growth can significantly alter the morphology.^38,39^ Additionally, while VLSM relies on a voxel-by-voxel statistical approach typically employing t-tests to compare behavioral scores between patients with and without lesions on each voxel, SVR-LSM utilizes support vector regression—a machine learning technique. This enables SVR-LSM to capture multiple variables and complex, nonlinear relationships between lesions and behavioral outcomes. Moreover, SVR-LSM excels at detecting subtle and nonlinear relationships between lesions and symptoms, thus offering a more nuanced understanding of the data that might be overlooked by the simpler statistical tests used in VLSM.^23,24,40^ In that regard, our work should be seen as complementary, rather than contradictory to the findings of Tejada Neyra et al., as it aims to uncover more subtle relationships between tumor localization and methylation class through the utilization of advanced analytical techniques. However, this heightened sensitivity could detect subtle yet clinically insignificant variations, affecting the results’ specificity. Therefore, future studies should balance sensitivity and specificity to ensure the robustness and clinical relevance of the findings. Tejada Neyra et al. did not consider FLAIR-hyperintense parts in contrast-enhancing tumors in their analysis. Our study adopted a unified approach using a single mask for all tumors, including the contrast-enhancing, FLAIR-hyperintense, and necrotic components. This decision was informed by studies indicating that the tumor is relevant beyond the limits of the contrast-enhancing regions.^41^ By avoiding a priori assumptions and excluding tumor compartments, we aimed to provide a holistic view of the tumor’s spatial characteristics.

Our study is subject to certain limitations that warrant consideration. First, the analysis is based on a monocentric dataset, necessitating validation of our findings across additional datasets to ensure generalizability. Despite this, it is noteworthy that our dataset, encompassing over 400 patients, is substantial in size relative to other studies focused on distinguishing methylation types in GB. Secondly, our investigation was confined to 3 specific methylation classes: MES, RTK I, and RTK II. It is important to highlight, however, that these classes represent the most prevalent methylation subclasses observed in the adult population.

In conclusion, our research has delineated the preferential localizations of various GB methylation subtypes through the application of SVR-LSM, offering valuable insights into the intricate relationship between GB’s molecular diversity and its spatial distribution within the brain. The identification of specific regions of predilection, particularly within the insula and frontal lobes, highlights the significant role that the brain’s unique metabolic and functional environments may play in tumorgenesis and progression. These findings not only deepen our understanding of GB pathophysiology but also suggest these localized areas as potential targets for therapeutic intervention in future research endeavors. Future studies should validate these findings in larger, independent cohorts to confirm the observed patterns.

## Data Availability

The data used in the present study is proprietary and confidential, belonging exclusively to the Heidelberg University Hospital. Due to the sensitive nature of this information and following our organization’s policies and confidentiality agreements, these data cannot be made available to third parties.
